# Mechanical Thrombectomy in Patients With Cervical Artery Dissection: A Multicenter Analysis on Technical, Safety, and Functional Outcomes

**DOI:** 10.1111/ene.70632

**Published:** 2026-06-22

**Authors:** Francesco Favruzzo, Marialuisa Zedde, Luca Weis, Rosario Pascarella, Alessia Giossi, Michele Besana, Francesco Valletta, Manuel Cappellari, Benedetto Petralia, Mauro Gentile, Andrea Zini, Luigi Simonetti, Stefano Vallone, Maria Giulia Mosconi, Maurizio Paciaroni, Andrea Fiacca, Aurelia Zauli, Pietro Caliandro, Iacopo Valente, Ludovica Ferraù, Paolo La Spina, Agostino Tessitore, Roberto Menozzi, Chiara Ferraro, Francesco Causin, Alessandro Pezzini, Claudio Baracchini

**Affiliations:** ^1^ Stroke Center and Neurosonology Laboratory Azienda Ospedale ‐ Università Padova Padua Italy; ^2^ Department of Neurology and Stroke Center Azienda Unità Sanitaria Locale‐IRCCS Di Reggio Emilia Reggio Emilia Italy; ^3^ IRCCS San Camillo Hospital Venezia Italy; ^4^ Department of Neuroradiology Ospedale Santa Maria Della Misericordia Rovigo Italy; ^5^ Department of Neurology and Stroke Center ASST Cremona Hospital Cremona Italy; ^6^ Department of Neuroradiology ASST Cremona Hospital Cremona Italy; ^7^ Department of Neurology and Stroke Center Azienda Ospedaliera Integrata, University of Verona Verona Italy; ^8^ Neurovascular Interventional Unit Azienda Ospedaliera Integrata, University of Verona Verona Italy; ^9^ Department of Neurology and Stroke Center IRCCS Istituto Delle Scienze Neurologiche Di Bologna, Maggiore Hospital Bologna Italy; ^10^ Neuroradiology Unit IRCCS Istituto Scienze Neurologiche Di Bologna, Maggiore Hospital Bologna Italy; ^11^ Department of Neuroradiology Ospedale Civile Di Baggiovara Modena Italy; ^12^ Cardiovascular and Emergency Medicine and Stroke Center Azienda Ospedaliera Santa Maria Della Misericordia, University of Perugia Perugia Italy; ^13^ Department of Neurosciences and Rehabilitation University of Ferrara Ferrara Italy; ^14^ Department of Neuroradiology Azienda Ospedaliera Santa Maria Della Misericordia, University of Perugia Perugia Italy; ^15^ Department of Neurosciences Università Cattolica Del Sacro Cuore Rome Italy; ^16^ Department of Neurology and Stroke Center Fondazione Policlinico Universitario A. Gemelli IRCCS Rome Italy; ^17^ Unit of Interventional Neuroradiology Fondazione Policlinico Universitario A. Gemelli IRCCS Rome Italy; ^18^ Department of Neurology and Stroke Center, Department of Experimental Biomedicine and Clinical Neurosciences University of Messina Messina Italy; ^19^ Neuroradiology Unit, Diagnostic Department University of Messina Messina Italy; ^20^ Department of Neuroradiology Parma University Hospital Parma Italy; ^21^ Department of Medicine and Surgery, University of Parma, Stroke Care Program, Department of Emergency Parma University Hospital Parma Italy; ^22^ Neuroradiology Unit Azienda Ospedale ‐ Università Padova Padua Italy

**Keywords:** acute ischemic stroke, carotid artery dissection, endovascular procedures, safety, treatment outcome, vertebral artery dissection

## Abstract

**Background and Aims:**

The safety and effectiveness of mechanical thrombectomy (MT) in patients with cervical artery dissection (CeAD) remain uncertain. This study aimed to evaluate the safety, recanalization rates, and functional outcomes of MT in LVO‐AIS patients with and without CeAD.

**Methods:**

Retrospective multicenter study based on data prospectively collected from June 2021 to June 2024. CeAD‐related LVO‐AIS patients treated with MT were compared with matched anterior and posterior circulation AIS patients without CeAD. Primary outcomes included procedural adverse events, recanalization rates, and favorable functional outcomes at 3 months. A meta‐analysis of similar studies from 2015 to 2025 was conducted to support generalizability.

**Results:**

Of 1861 LVO‐AIS patients, 164 (8.7%) patients had a CeAD. Despite a higher prevalence of tandem occlusion (63.0% vs. 20.0%, SDM: 0.99), CeAD patients showed similar rates of overall procedural adverse events (17.7% vs. 16.3%, *p* = 0.684), recanalization rates (90.0% vs. 87.5%, *p* = 0.563), and 90‐day favorable functional outcome (59.1% vs. 58.5%, *p* = 0.917) as compared to non‐CeAD patients. Additionally, CeAD patients had a significantly lower mortality rate at 90 days (4.3% vs. 13.1%, *p* = 0.023). A subgroup analysis of posterior circulation LVO‐AIS showed no significant differences in safety and clinical outcomes. Meta‐analysis of six studies suggested better MT outcomes in CEAD‐related LVO‐AIS (*p* < 0.001, with *I* ^2^ = 60.02% [13.48–83.33 95% CI]).

**Conclusions:**

Despite procedural challenges, this multicenter study has shown that in both anterior and posterior circulation CeAD‐related LVO‐AIS, MT is as safe as in non‐CeAD stroke patients, achieves high recanalization rates, and is associated with similar favorable functional outcomes.

## 1 Introduction

Mechanical thrombectomy (MT) is the most effective therapy for acute ischemic stroke (AIS) patients with large vessel occlusion (LVO), as it improves survival and reduces disability [1, 2].

Yet, cervical artery dissection (CeAD), which accounts for 2% of AIS and up to 25% of AIS in young adults, was underrepresented in the thrombectomy trials, as it often poses technical challenges for neuro‐interventionists due to a high occurrence of tandem occlusions and concerns about arterial wall weakness [3, 4], resulting in longer procedure time and potentially higher adverse event rates, as often one or more stents are deployed [5]. Consequently, data on MT outcomes in CeAD remain limited, raising the question of whether its safety and efficacy differ from other stroke etiologies. Current evidence regarding MT in CeAD patients is primarily derived from a limited number of observational studies [6, 7, 8, 9, 10]. The American Heart Association and European Stroke Organization recommend MT as a reasonable option for CeAD‐related LVO, acknowledging the lack of CeAD‐specific outcomes and safety data [4, 11]. Notably, posterior circulation LVOs are not specifically addressed, leaving uncertainty regarding treatment in this territory [11]. Hence, this multicenter study aimed to evaluate the safety, recanalization rates, and functional outcomes of MT in a large cohort of CeAD‐related LVO strokes compared to non‐CeAD LVO stroke patients. Furthermore, we performed a subgroup analysis of posterior circulation LVOs and a systematic review and meta‐analysis to provide a cumulative estimate of the treatment effect of MT in CeAD patients.

## 2 Patients and Methods

### 2.1 Design and Population

This Italian retrospective multicenter observational study used prospectively collected data from LVO‐AIS patients who underwent MT between June 2021 and June 2024 at nine participating stroke centers with expertise in CeAD management (see Figure S1 for contributing centers) [12]. All registry data were reviewed locally by trained neuroradiologists and neurologists and underwent a centralized quality check for uniformity. Eligibility criteria were: (a) patients ≥ 18 years; (b) LVO‐AIS, eligible for MT; and (c) available 90‐day mRS score.

For the purpose of this study, the population was divided into two groups: CeAD‐related stroke patients and stroke patients due to other etiologies. Broadly accepted diagnostic criteria were applied for CeAD diagnosis at each center: the presence of a mural hematoma, a long tapering stenosis, an intimal flap, a double lumen, or an occlusion situated 2 cm above the carotid bifurcation, revealing an aneurysmal dilation (pseudoaneurysm) [13, 14]. For the non‐CeAD group, stroke etiology was determined according to the Trial of Org 10,172 in Acute Stroke Treatment (TOAST) classification at each center [15].

Patients with intracranial, traumatic, iatrogenic, or common carotid artery dissections were excluded to ensure a more homogeneous study population and to improve the interpretability of the results.

Patient care adhered to national [16] and international [17] guidelines for intravenous thrombolysis (IVT) and MT eligibility.

The study protocol was approved by the local institutional review boards of each participating center. The STrengthening the Reporting of Observational studies in Epidemiology (STROBE) guidelines were followed for this study [18].

### 2.2 Data Extraction

Data were retrieved from each site and analyzed centrally (see Figure S1 for the full list of variables extracted).

### 2.3 Endovascular Treatment

The choice of the endovascular technique and anesthesia type adopted was at the discretion of the neuro‐interventionist, based on experience, complexity, and patient characteristics. General anesthesia was primarily used in patients with poor general conditions (e.g., reduced consciousness, agitation, severe cardiovascular or respiratory impairment, persistent vomiting, tandem occlusion). Each procedure employed a system of balloon guide catheter and microcatheter introduced via transfemoral or transradial access. If hypoperfusion was detected distal to a severe stenosis or occlusion of a cervical vessel, one or more stents were deployed along the affected segment. The choice of approach was also chosen by the neuro‐interventionist: anterograde (opening the extracranial dissected vessel first, then the intracranial LVO) or retrograde (opening the intracranial LVO first, then securing the cervical lesion). Intracranial recanalization was assessed using the modified Treatment in Cerebral Infarction (mTICI) score. Dual antiplatelet therapy was administered to all stented patients; for non‐stented patients, the choice of secondary prevention was determined by experienced vascular neurologists.

### 2.4 Study Outcomes

Regarding safety, the primary endpoints included the incidence of adverse events during or after the endovascular procedure (detailed definition of each adverse event is provided in the Figure S1). The primary neuroradiological endpoint was the degree of recanalization achieved by MT; namely, a successful procedure was defined as mTICI score 2b, 2c, or 3. The main clinical outcomes were functional independence, defined as a favorable modified Rankin Scale (mRS ≤ 2), and mortality rates at 90 days from AIS onset. Outcomes were assessed and recorded at each participating center by experienced neuroradiologists/neurologists.

### 2.5 Covariate Selection and Propensity Score Matching

Covariates relevant to outcome prediction were selected based on clinical relevance and literature evidence to enhance the generalizability and robustness: Age, sex, pre‐stroke mRS, baseline NIHSS, and diabetes [19, 20, 21]. These covariates were integrated into the models together with those showing a significant impact on 90‐day mRS in our whole sample to ensure adequate adjustment for confounders.

For the data‐driven approach, baseline characteristics were compared between patients with favorable (mRS 0–2) and unfavorable (mRS > 2) outcomes using univariate logistic regression or Mann–Whitney *U* tests, as appropriate. To minimize bias from imputation, the primary outcome analysis was conducted using a complete‐case dataset restricted to individuals with non‐missing values.

Propensity score matching (PSM) was performed using a weighted distance metric (1.5 × Mahalanobis for continuous and 0.9 × Hamming for binary variables) in a 1:1 ratio. Matching covariates were chosen based on clinical relevance and literature support. Weighting parameters were iteratively optimized to minimize the overall median standardized difference across variables. Missing data were imputed for exploratory modeling; however, PSM was restricted to complete cases to ensure comparability. Multicollinearity was assessed using variance inflation factors (VIF), and only predictors with VIF < 5 were retained to preserve model stability.

### 2.6 Meta‐Analysis

To evaluate the safety and functional outcomes of MT in CeAD patients across the literature, a systematic literature search was conducted in February 2025 across PubMed, Embase, Medline, the Web of Science Core Collection, and the Cochrane Library. The search included studies published between January 2015 and February 2025. Search strategies included terms regarding the study population (e.g., *‘human’, ‘stroke’, ‘ischemic stroke’, ‘brain infarction’, ‘cerebrovascular disorder’, ‘Cervical artery dissection’*), intervention (e.g., *‘Endovascular treatment’, ‘Mechanical thrombectomy’, ‘endovascular thrombectomy’, and ‘endovascular surgery’*), and outcomes (e.g., *‘functional outcome’, ‘modified Rankin scale’*). Variations of these terms were applied to ensure comprehensive retrieval. Search strategies combined controlled vocabulary (MeSH terms for MEDLINE and Emtree terms for Embase) with free‐text keywords searched in titles, abstracts, and author keywords, and were linked using Boolean operators. Strategies were adapted for each database. Embase (Ovid) was searched from January 1, 2015, to February 15, 2025, using the Advanced Search command‐line interface. The strategy combined controlled Emtree vocabulary (exploded) with free‐text terms. Records from the Cochrane Central Register of Controlled Trials (CENTRAL) available within the Embase Ovid platform were also included. Boolean operators were used to combine concepts relating to ischemic stroke, cervical artery dissection, and endovascular treatment. Limits for human studies, adult populations, and the English language were applied after combining concepts. Eligible study designs included randomized controlled trials and non‐randomized cohort studies. Full search strategies are provided in the Figure S1 to ensure reproducibility, in accordance with Cochrane recommendations for reporting electronic searches. Inclusion criteria required comparison of MT outcomes in CeAD and non‐CeAD patients, publication within the specified timeframe, and reporting at least one predefined outcome of interest. Exclusion criteria were: Absence of clinical outcome data, lack of a control group, studies on limited stroke etiologies, unavailable full‐text format, case reports, or sample size < 10 patients to avoid anecdotal bias. Two investigators independently reviewed the results for eligibility. Risk of bias was assessed with the Risk of Bias in Non‐randomized Studies—of Interventions, Version 2 (ROBINS‐I V2) tool, and visualization was generated using the Robvis tool [22]. The review protocol was registered in PROSPERO (CRD420251037840).

### 2.7 Statistical Analysis

Categorical variables were summarized as counts and percentages; continuous variables as mean ± standard deviation or median with interquartile range, depending on distribution. Balance between CeAD and non‐CeAD groups was evaluated using standardized mean differences (SMD).

For continuous variables:

(1) $$\mathrm{SMD}\_\text{continuous}=\left({\bar{X}}_{1}-{\bar{X}}_{2}\right)/\text{sqrt}\left(\left({s_{1}}^{2}+{s_{2}}^{2}\right)/2\right).$$

where X–_1_ and X–_2_ are the group means, and s_1_^2^ and s_2_^2^ are the group variances.

For binary variables:

(2) $$\mathrm{SMD}\_\text{binary}=\left(p_{1}-p_{2}\right)/\text{sqrt}\left(p\left(1-p\right)\right),\text{where}\;p=\left(p_{1}+p_{2}\right)/2.$$

with p_1_ and p_2_ representing the proportions in each group.

Interpretation of SMD values followed conventional thresholds: < 0.1, negligible difference, 0.1–0.2, small difference, 0.2–0.5, moderate difference, and > 0.5 relevant imbalance. Covariate balance was visualized with a Love plot.

Differences in binary outcomes between subgroups were analyzed using logistic regression; continuous outcomes were analyzed using generalized linear models. Both were adjusted for confounders. Adjusted odds ratios (aOR) with 95% confidence intervals (CI) were calculated, and ORs for CeAD vs. matched non‐CeAD patients were visualized via a forest plot.

Regarding meta‐analysis, both random‐ and fixed‐effects analyses were applied to estimate pooled effects, displayed in a forest plot. Heterogeneity was quantified with the *I* ^2^ test: 25%–74% indicating moderate, ≥ 75% substantial heterogeneity [23].

All statistical analyses were conducted using SPSS v.25.0, except PSM, which used the Sklearn.neighbors library in Python 3.9.18, and multiple imputation with the missRanger package in R 4.3.2 library. Two‐tailed *p* < 0.05 was considered significant.

## 3 Results

### 3.1 Baseline Characteristics

A total of 1878 patients were eligible for this study; 17 were then excluded due to missing values for a total of 1861 patients: 164 presented with LVO‐AIS attributable to spontaneous CeAD, while 1697 had other stroke etiologies (see Figure S2).

Table 1 presents the baseline characteristics of the entire cohort and the two groups stratified by etiology. Overall, the median age of the study population was 75 years (64–82), 50% were females. At presentation, 91% of patients had anterior circulation LVO strokes with a median NIHSS score of 15 (10–19), and 41% received IVT. Among non‐CeAD patients, the most represented site of occlusion was the middle cerebral artery (63%, see Figure S3), whereas the most frequent stroke etiology was cardioembolic (48.7%), followed by large vessel atherosclerosis (21.3%), stroke of undetermined cause (20.0%), and stroke of other determined cause (10.0%). Among CeAD patients, the most frequent site of dissection was the ICA (92.7%).

**TABLE 1:** Baseline characteristics of the study population.

	Overall population *N* = 1,875			CeAD *N* = 164			Non‐CeAD *N* = 1,697			PSM–Non‐CeAD *N* = 160			CeAD/Non‐CeAD StdDiff	CeAD/PSM Non‐CeAD StdDiff
	*N*	Median/%	IQR	*N*	Median/%	IQR	*N*	Median/%	IQR	*N*	Median/%	IQR		
Clinical and demographic characteristics														
Age	1861	75	64–82	164	54	46 – 60	1697	76	67–83	160	54	46–60	**1.57** ^**b**^	0.03
Sex (female)	1861	50%		164	40%		1697	51%		160	41%		0.23^a^	0.04
Baseline mRS	1855	0	0–1	164	0	0–0	1691	0	0–1	160	0	0–0	**0.65** ^**b**^	0.00
Baseline NIHSS	1847	15	10–19	162	15	8–18	1685	15	10–19	160	13	7–18	**0.58** ^**b**^	**0.66** ^**b**^
Onset‐to‐groin time (min.)	1467	235	155–290	164	180	119–270	1303	237	159–290	160	192	133–279	0.08	0.03
Last known well–to‐door time (min.)	1481	120	67–240	142	110	65–239	1339	120	67–240	145	120	75–302	0.08	0.09
ASPECTs	1421	10	9–10	153	10	9–10	1268	10	9–10	160	9	8–10	0.03	0.05
Occluded vessel (Ant. circulation)	1839	91%		164	93%		1675	92%		160	88%		0.07	0.17
Mothership	1859	42%		164	45%		1695	42%		160	40%		0.08	0.09
Tandem occlusion	1797	24%		163	63%		1634	20%		157	22%		**0.99** ^**b**^	**0.92** ^**b**^
Thrombolysis	1833	41%		164	37%		1669	42%		159	40%		0.21^a^	0.22^a^
Risk factors														
Absence of all risk factors	1861	7%		164	24%		1697	5%		160	14%		**0.57** ^**b**^	0.29^a^
Smoking habits	1820	22%		161	29%		1659	21%		157	28%		0.24^a^	0.05
Alcohol consumption	1792	38%		151	20%		1636	40%		153	33%		0.46^a^	0.36^a^
Arterial hypertension	1861	69%		164	38%		1697	72%		160	49%		**0.73** ^**b**^	0.24^a^
Diabetes mellitus	1869	18%		164	7%		1697	19%		160	12%		0.35^a^	0.18
Cardiovascular disease	1861	7%		164	4%		1697	7%		160	7%		0.16	0.15
Dyslipidemia	1860	38%		164	31%		1696	39%		160	30%		0.16	0.02
Atrial fibrillation	1861	32%		164	10%		1697	35%		160	19%		**0.63** ^**b**^	0.25^a^
Cerebrovascular disease	1861	7%		164	4%		1697	7%		160	7%		0.16	0.15
Respiratory diseases	1860	5%		164	1%		1696	5%		160	6%		0.22^a^	0.25^a^
Renal insufficiency	1858	3%		164	0%		1694	4%		159	1%		0.29^a^	0.16
Hematologic disease	1860	2%		164	2%		1696	2%		160	3%		0.05	0.00
Oncological diseases	1859	9%		164	3%		1695	10%		160	7%		0.29^a^	0.20
Liver insufficiency	1859	0%		164	0%		1695	1%		159	1%		0.11	0.16
Pharmacological therapy														
Antihypertensive therapy	1859	55%		164	26%		1695	58%		160	33%		**0.67** ^**b**^	0.16
Lipid‐lowering therapy	1858	27%		164	16%		1694	28%		160	18%		0.30^a^	0.05
Urate‐lowering therapy	1859	5%		164	1%		1695	5%		160	3%		0.23^a^	0.13
Hypoglycemic therapy	1860	14%		164	2%		1696	15%		160	11%		0.47^a^	0.34^a^
Antiinflammatory therapy	1859	3%		163	1%		1696	3%		160	3%		0.17	0.17
Respiratory therapy	1860	2%		164	1%		1696	2%		160	1%		0.08	0.00
Antithrombotic therapy	1858	44%		164	17%		1694	47%		160	27%		**0.67** ^**b**^	0.23^a^

*Note:* Covariate balance improvement between CeAD and Non‐CeAD groups before and after PSM Matching: < 0.1, negligible difference; 0.1–0.2, small difference. Bold values indicates relevant standardized mean differences.

Abbreviations: ASPECTS, Alberta Stroke Program Early CT Score; IQR, interquartile range; MRS, modified Rankin Scale; NIHSS, National Institutes of Health Stroke Scale; StdDiff, standardized mean differences.

^a^ 0.2–0.5, moderate difference.

^b^ > 0.5, relevant difference.

Compared to the control group, CeAD patients were younger (median age 54 vs. 76 years, SDM: 1.57), more functionally independent at stroke onset [median baseline mRS 0 (0.0–0.0) vs. 0 (0.0–1.0), SDM: 0.65], and exhibited lower rates of cardiovascular risk factors, except for smoking habit, which was more prevalent in the CeAD group (29.0% vs. 21.0%, SDM: 0.24). Additionally, CeAD patients were less likely to be taking antithrombotic, antihypertensive, or hypoglycemic medications prior to admission. The occurrence of tandem occlusion was higher in the CeAD group (63.0% vs. 20.0%, SDM: 0.99), reflecting a greater clinical stroke severity (median NIHSS score 15 [10, 11, 12, 13, 14, 15, 16, 17, 18, 19] vs. 13 [9, 10, 11, 12, 13, 14, 15, 16, 17, 18], SDM: 0.58). After applying PSM, the control group differed significantly from the CeAD group only in the prevalence of tandem occlusion (22.0% vs. 63.0%, SDM: 0.92) and stroke severity (median NIHSS score 15 [10, 11, 12, 13, 14, 15, 16, 17, 18, 19] vs. 13 [7, 8, 9, 10, 11, 12, 13, 14, 15, 16, 17, 18], SDM: 0.66) (see Figure S4).

Looking for confounders, multivariable analysis identified several variables associated with increased risk of poor functional outcome (mRS 3–6) (see Table S1). These were therefore utilized to correct analyses on study outcomes.

### 3.2 Safety Outcomes and Procedural Characteristics

CeAD patients were associated with longer groin‐to‐recanalization times (aB = 11.91, SE = 4,02, *p* = 0.003) and were more likely to require conversion to general anesthesia during the endovascular procedure (aOR 2.398, 95% CI 0.98–5.86, *p* = 0.050) compared to the overall non‐CeAD group (see Table S2). However, these differences were balanced in the matched group. Instead, the logistic regression model showed that intravenous and intra‐arterial drug administration other than IVT (aOR 3.777, 95% CI 2.10–6.79, *p* = 0.006 and aOR 2.496, 95% CI 1.29–4.79, *p* < 0.001) and cervical stent placement (aOR 4.573, 95% CI 2.41–8.69, *p* < 0.001) were more frequent in the CeAD group than in the matched control group.

Regarding adverse events, the cumulative incidence was similar between the two groups (aOR 1.139, 95% CI 0.61–2.14, *p* = 0.684). However, CeAD patients showed greater odds of distal embolization (OR 4.286, 95% CI 1.07–17.09, *p* = 0.039). Notably, the rates of other adverse events were similar between CeAD and non‐CeAD patients (see Table 2).

**TABLE 2:** Functional and safety outcomes of MT in CeAD and matched non‐CeAD patients.

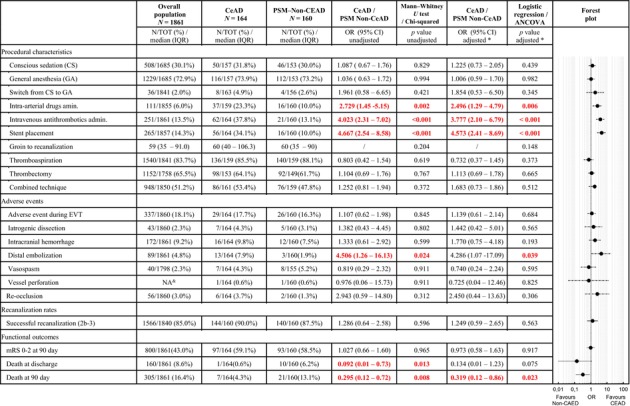

*Note:* Bold values indicates relevant standardized mean differences.

Abbreviations: IQR, interquartile range; mRS, modified Rankin Scale; mTICI, modified Treatment in Cerebral Infarction; MT, mechanical thrombectomy; NIHSS, National Institutes of Health Stroke Scale; OR, odds ratio.

* Multivariable logistic regression and Ancova models were adjusted for Age, Baseline mRS, Gender, baseline NIHSS, absence of risk factor, Smoking habits, Arterial hypertension, Diabetes mellitus, Atrial fibrillation, Antihypertensive therapy, Hypoglycemic therapy, and Antithrombotic therapy & Data collected for CeAD and PSM Non‐CeAD groups only.

### 3.3 Recanalization Rates

Successful recanalization rates were high in both groups, with no significant difference (90.0% vs. 87.5%, *p* = 0.563). Overall, recanalization rates did not differ significantly between CeAD and non‐CeAD patients (mTICI 0: 3.1% vs. 6.2%; mTICI 1: 2.5% vs. 1.2%; mTICI 2a: 4.4% vs. 5.8%; mTICI 2b: 21.6% vs. 16.7%; mTICI 2c: 15.0% vs. 9.6%; mTICI 3: 53.1% vs. 64.7%, *χ* ^2^(4) = 7.169, *p* = 0.21).

### 3.4 Functional Outcomes

CeAD patients showed better functional outcomes at 90 days than non‐CeAD patients in the unadjusted multivariate logistic regression model (OR 2.041, 95% CI 1.47–2.83, *p* < 0.001; see Table S2). However, after adjusting for confounders, CeAD patients exhibited similar rates of good clinical outcome compared to both overall (aOR 0.939, 95% CI 0.63–1.40, *p* = 0.756) and matched (aOR 0.973, 95% CI 0.58–1.63, *p* = 0.917) non‐CeAD groups.

**FIGURE 1:**
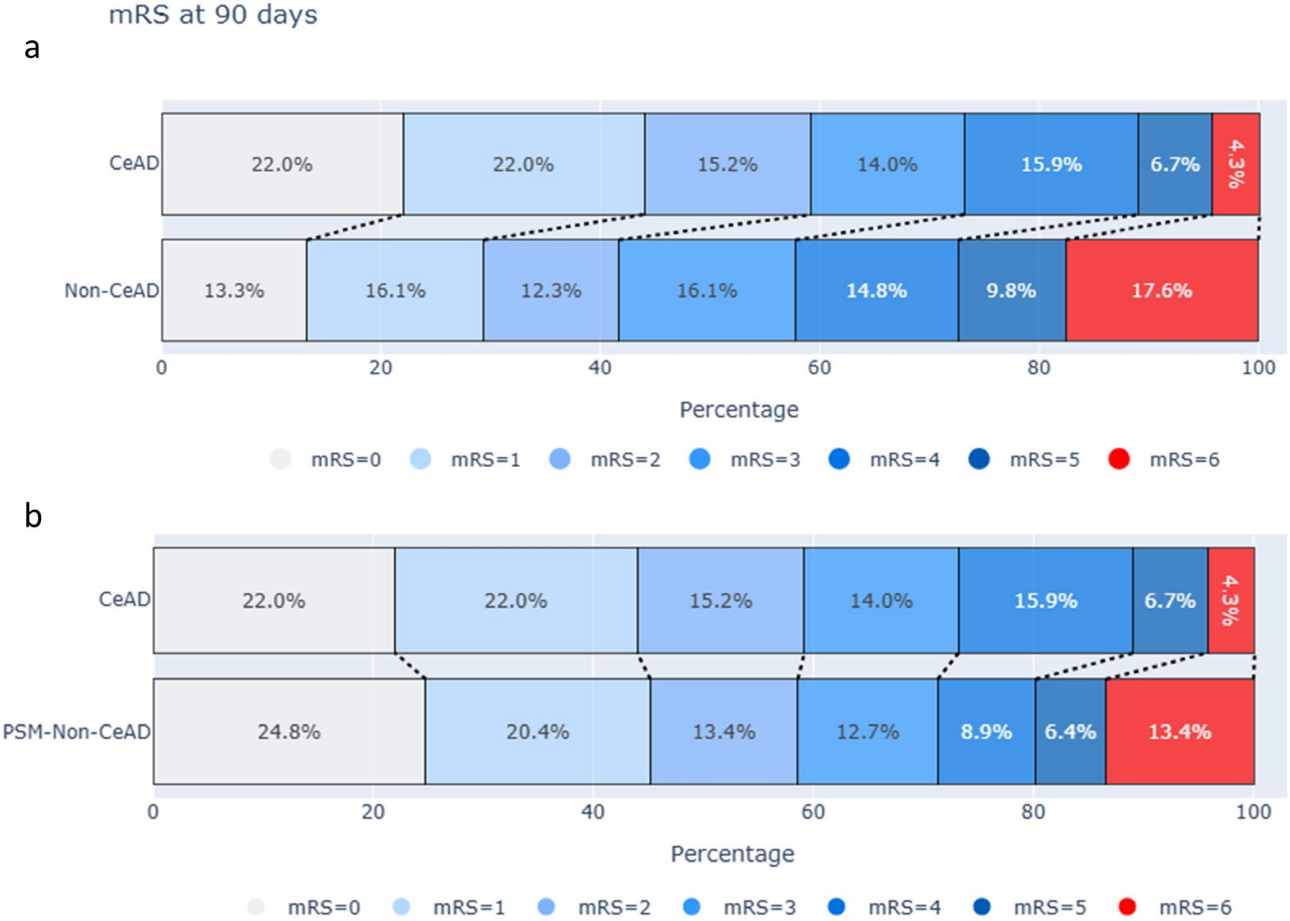
Comparison of functional outcomes and mortality rates at 90 days between (a) CeAD and overall non‐CeAD patients and (b) CeAD and matched non‐CeAD patients.

In both unadjusted and adjusted multivariable analyses, CeAD was associated with a significantly lower likelihood of death compared to both the overall and matched non‐CeAD groups at 90 days (aOR 0.408, 95% CI 0.18–0.92, *p* = 0.031; aOR 0.319, 95% CI 0.12–0.86, *p* = 0.023, respectively) and a lower likelihood of death compared to the overall non‐CeAD patients at discharge (aOR 0.098, 95% CI 0.01–0.73, *p* = 0.023). Results are summarized in Table 2 and displayed in Figure 1.

Within the CeAD group, logistic regression identified lower baseline mRS (OR 0.220, 95% CI 0.297 − 2.735; *p* = 0.015), lower baseline NIHSS score (OR 0.873, 95% CI 0.076 − 0.196; *p* < 0.001), and IVT administration (OR 2.653, 95% CI 0.205 – 1.746; *p* = 0.013) as predictors of good functional outcome at 90 days (see Table 3).

**TABLE 3:** Predictors of favorable functional outcomes within the CeAD group.

Logistic regression	Independent variable	OR	95% CI	*p*
90‐day mRS ≤ 2	Baseline NIHSS score	0.873	0.076 − 0.196	< 0.001
	Baseline mRS	0.220	0.297 − 2.735	0.015
	IVT	2.653	0.205 – 1.746	0.013

Abbreviations: 95% CI, 95% confidence interval; IVT, intravenous thrombolysis; mRS, modified Rankin Scale; NIHSS, National Institutes of Health Stroke Scale; OR, odds ratio.

These results remained consistent even after the additional adjustment for baseline ASPECTs and intracranial occlusion site, showing that these variables did not meaningfully alter the direction or magnitude of the association between CeAD status and functional outcomes (see Table S3).

Moreover, to test a possible residual age‐related confounding effect, an additional exploratory analysis was conducted examining age distributions in relation to the two primary clinical endpoints: function independence and mortality. As shown in Figure S5, an independent *t*‐test demonstrated no significant age differences between the corresponding matched subgroups.

### 3.5 Posterior Circulation Analysis

Twelve out of 164 (7.3%) CeAD patients and 19 out of 160 (11.9%) matched non‐CeAD patients presented with a posterior circulation LVO. The CeAD group had a lower median age than the control group (49.5 vs. 58.0, SDM: 0.88), lower baseline NIHSS score (12.0 vs. 14.0, SDM: 3.12), and lower onset‐to‐door time (74.0 vs. 104.0, SDM: 1.10) (see Table S4).

No significant differences were observed in mortality rates, functional outcomes at 90 days, or recanalization rates (Table 4).

**TABLE 4:** Functional and safety outcomes of sensitivity analysis for CeAD and matched non‐CeAD patients with posterior circulation LVO.

	Overall population	CeAD *N* = 12	PSM Non‐CeAD *N* = 19	CeAD/PSM Non‐CeAD	Mann–Whitney/Fisher exact test
	*N*/TOT (%)/median (IQR)	*N*/TOT (%)/median (IQR)	*N*/TOT (%)/median (IQR)	OR (95% CI)	*p*
Procedural characteristics					
Conscious sedation (CS)	9/30 (30.0%)	3/11 (27.3%)	6/19 (31.6%)	0.812 (0.16–4.19)	1
General anesthesia (GA)	22/30 (73.3%)	9/11 (81.8%)	13/19 (68.4%)	2.077 (0.34–12.7)	0.672
Switch from CS to GA	2/30 (6.7%)	1/12 (8.3%)	1/18 (5.6%)	1.546 (0.09–27.36)	1
Intra‐arterial drugs admin.	3/31 (9.7%)	3/12 (25.0%)	0/19 (0%)	14.368 (0.67–307.37)	**0.048**
Intravenous antithrombotics admin.	6/31 (19.4%)	4/12 (33.3%)	2/19 (10.5%)	4.25 (0.69–28.25)	0.174
Stent placement	5/31 (16.1%)	3/12 (25.0%)	2/19 (10.5%)	2.83 (0.39–20.18)	0.350
Groin to recanalization	45 (26.5–71.3)	55.5 (41.0–65.0)	40 (18–78.8)	NA	0.249
Thromboaspiration	29/31 (93.5%)	11/12 (91.7%)	18/19 (94.7%)	0.611 (0.03–10.80)	1
Thrombectomy	19/30 (63.3%)	8/12 (66.7%)	11/18 (61.1%)	1.273 (0.28–5.87)	1
Combined technique	17/31 (54.8%)	7/12 (58.3%)	10/19 (52.6%)	1.260 (0.29–5.41)	0.100
Adverse events					
Adverse event during MT	3/31 (9.7%)	2/12 (16.7%)	1/19 (5.3%)	3.60 (0.29–44.83)	0.543
Iatrogenic dissection	0/31 (0%)	0/12 (0%)	0/19 (0%)	NA	/
Intracranial hemorrhage	2/31 (6.5%)	2/12 (16.7%)	0/19 (0%)	9.29 (0.41–211.92)	0.142
Distal embolization	1/31 (3.2%)	0/12 (0%)	1/19 (5.3%)	0.49 (0.02–13.11)	**1**
Vasospasm	0/30 (0%)	0/12 (0%)	0/18 (0%)	NA	/
Re‐occlusion	0/31 (0%)	0/12 (0%)	0/19 (0%)	NA	/
Recanalization rates					
Successful recanalization (2b‐3)	26/29 (89.7%)	11/12 (91.7%)	15/17 (88.2%)	1.467 (0.12–18.29)	1
Functional outcomes					
mRS 0–2 at 90 day	16/31 (51.6%)	5/12 (41.7%)	11/19 (57.9%)	0.606 (0.14–2.71)	0.707
Death at 90 day	5/31 (16.1%)	1/12 (8.3%)	4/19 (21.1%)	0.341 (0.03–3.49)	0.624

*Note:* Bold values indicates relevant standardized mean differences.

Abbreviations: IQR, interquartile range; mRS, modified Rankin Scale; mTICI, modified Treatment in Cerebral Infarction; MT, endovascular treatment/mechanical thrombectomy; NIHSS, National Institutes of Health Stroke Scale; OR, odds ratio.

Regarding safety, both groups showed comparable adverse events associated with MT. However, CeAD patients required intra‐arterial drug administration more frequently than non‐CeAD patients (25.0% vs. 0.0%, *p* = 0.048). No other significant differences in procedural characteristics were identified.

Importantly, given the distinct clinical presentation and prognosis of posterior circulation strokes, a sensitivity analysis restricted to patients with anterior circulation LVO was performed (see Table S5). The results confirmed the findings observed in the overall cohort.

### 3.6 Meta‐Analysis

**FIGURE 2:**
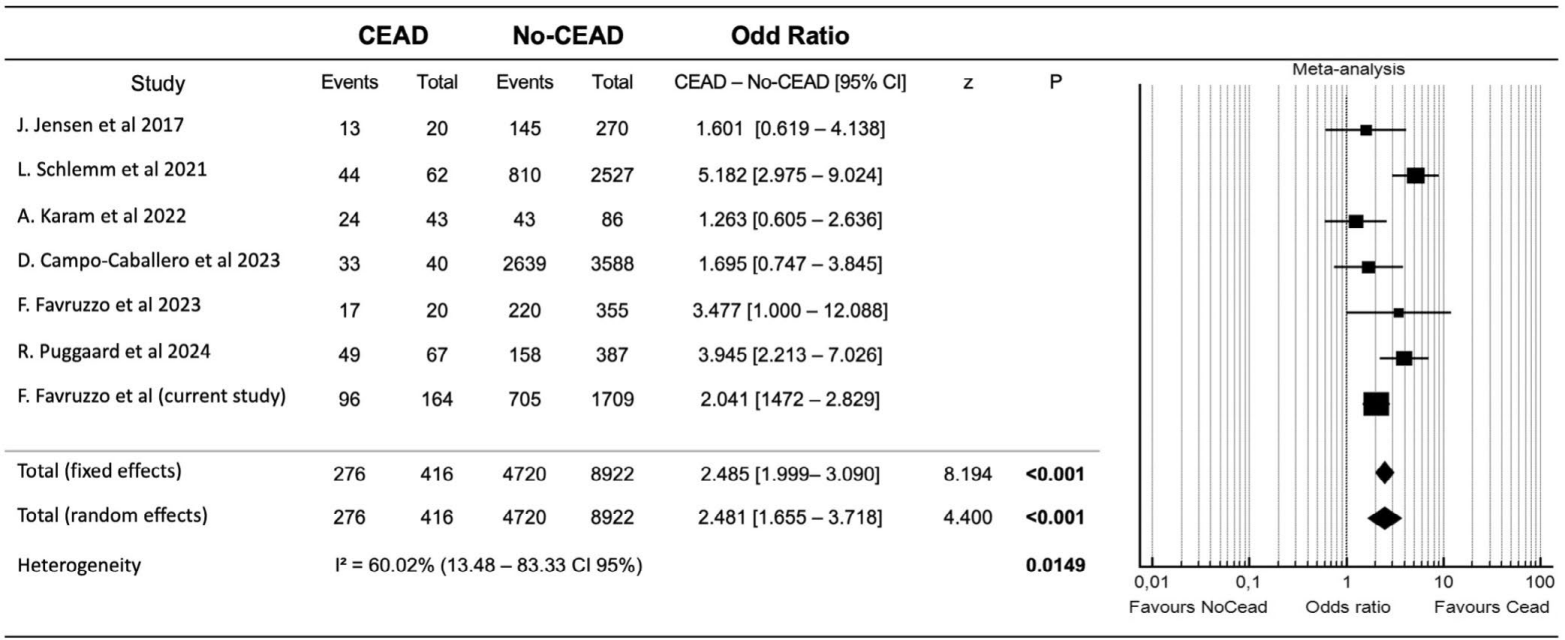
Results from meta‐analysis showing the OR with the corresponding 95% CI of every single study and the overall calculated OR for the estimate of the total effect.

From a total of 765 articles, six articles were finally selected (the detailed PRISMA flow chart is available in Figure S6) [7, 24, 25, 26, 27, 28]. These included five retrospective studies and one propensity‐matched retrospective study, collectively involving 7465 patients (252 CeAD and 7213 non‐CeAD patients). The overall ORs for good functional outcomes between the two groups, derived from logistic regression models after including the current work, are presented in Figure 2. Moderate heterogeneity was observed (*I* ^2^ = 60.02%), supporting the use of the random‐effects model as the primary analysis. However, either using a fixed‐effects model [aOR 2.226 (95% CI 1.801–2.752, *p* < 0.001)] or a random‐effects model aOR 2.318 (95% CI 1.287–4.175, *p* = 0.005), the pooled OR was always in favor of CeAD patients, indicating a moderate positive effect of MT in the dissected patients. The overall risk of bias was moderate to serious in all the studies analyzed (see Figure S7 and Figure S8).

## 4 Discussion

This large multicenter study revealed that while CeAD represents a procedural challenge for neuro‐interventionists, MT demonstrates comparable safety and yields similar clinical and neuroradiological outcomes to those observed in matched patients with other stroke etiologies in both anterior and posterior circulation LVO‐AIS. Moreover, dissected patients demonstrated significantly lower mortality rates.

The main procedural concern when performing MT in CeAD‐related LVO‐AIS is the high occurrence of tandem occlusions [3]. Accordingly, in our cohort, the rate was three times higher in CeAD patients than in the control group (63% vs. 20%, *p* < 0.01), likely explaining the greater clinical severity and aligning with previous reports [25, 28]. This significant difference in tandem occlusions may be attributable to the pathophysiology of CeAD itself, where endothelial dysfunction frequently leads to high‐grade stenosis or occlusion of the dissected vessel [29], and a poorer prognosis [30]. Moreover, the residual NIHSS imbalance at presentation may also reflect underlying pathophysiological differences rather than inadequate matching: concomitant occlusion of the ipsilateral cervical artery, together with the lower burden of traditional cardiovascular risk factors, is likely associated with poorer cerebral collateral circulation or reduced perfusion pressure in the territory at infarction, resulting in more severe clinical deficits at presentation.

Notably, the median ASPECT score was similar in the two groups, possibly reflecting the earlier CT scan acquisition and a lower burden of cardiovascular disease in CeAD patients.

With regards to the safety of MT in CeAD patients, we analyzed the following adverse events: iatrogenic dissection, intracranial hemorrhage, distal embolization, vasospasm, and re‐occlusion of the recanalized segment. No significant differences were observed in the cumulative incidence of the events; however, CeAD carries higher odds of periprocedural distal embolization. Notably, this difference may be linked to the frequent carotid lesion in this group and the consequently neuro‐interventionalist's caution when navigating a dissected cervical vessel to avoid extension [31], together with the greater rate of periprocedural stenting, possibly dislodging emboli distally. The increased administration of drugs in the CeAD cohort, on the other hand, further supports the notion of a more complex intervention in dissected patients, often necessitating conversion from conscious sedation to general anesthesia.

Despite the aforementioned technical challenges, intracranial recanalization rates did not differ significantly between the two groups. Overall, MT was successful in about 90% of cases, consistent with previous works analyzing MT [24, 25, 26, 27, 28]. A slight superiority was observed in the CeAD group, possibly related to the higher rate of emergent stent placement, as suggested by the recent secondary analysis of the STOP‐CAD study [32]. This translated into comparable rates of functional independence at three months and significantly lower mortality in CeAD patients. Surely, an excellent finding, given the procedural difficulties of MT and the potentially worse prognosis of CeAD patients with tandem occlusion [30]. Moreover, a novel result is represented by the lower mortality rate observed in CeAD patients compared to non‐CeAD patients of similar age. Although this might be related to sample size, it could also reflect the worse cardiovascular risk profile of non‐CeAD patients.

The neuroradiological and functional outcomes documented in this study encourage neurologists and neuro‐interventionalists to consider MT when managing LVO in the context of CeAD. The rates of successful recanalization and favorable clinical outcomes in our cohort are also in line with two recently published papers analyzing MT in CeAD patients during the same time period as the present study. Specifically, the rates of good clinical outcomes at 90 days in CeAD patients undergoing EVT ranged from 58.5% to 62.0%, compared with only 43.9% in those receiving best medical treatment [10, 32]. However, in contrast to those studies, which exclusively focused on CeAD populations, our work offers a direct comparison between CeAD and non‐CeAD patients undergoing MT. This approach provides additional insight that better reflects real‐world experience, especially regarding the procedural complexity and safety of MT in CeAD. Additionally, our results indicate that IVT is an independent predictor of good functional outcomes in CeAD patients, reinforcing its beneficial effects in this population, as demonstrated in previous studies [33]. These findings suggest that bridging therapy may be the optimal treatment also for LVO‐AIS related to this etiology.

Our meta‐analysis consolidates findings from six retrospective analyses comparing MT outcomes in CeAD vs. non‐CeAD patients. A total of 252 CeAD patients were added to our cohort, resulting in a final sample size of 416 cases. The pooled analysis reinforces our primary finding that CeAD patients achieve comparable functional outcomes to other patients regardless of increased procedural challenges, with a higher likelihood of functional independence. Notwithstanding a moderate heterogeneity, reflecting real‐world variability in procedural characteristics and AIS management across studies, the odds of functional independence were nearly 2.5 times higher for CeAD patients than for those with other stroke etiologies. This cumulative estimate highlights a significant benefit of MT for LVO due to CeAD. The consistency of these results across different populations and study designs strengthens the overall validity and generalizability of our findings, indicating that the neutral results observed in our large multicenter cohort are not isolated but are concordant with—and, in aggregate, slightly favor—outcomes in CeAD‐related LVO‐AIS reported in the existing literature, despite substantial clinical heterogeneity.

Greater uncertainty exists regarding the effect of MT on CeAD patients with posterior circulation LVO, as no recommendations are available in international guidelines [4, 11]. To date, only two retrospective studies have included dissected patients who underwent MT for posterior circulation LVO, totaling 26 cases [25, 28]. The exploratory sensitivity analysis from our cohort, although limited by a relatively small number of patients (*n* = 12) and the younger age of the CeAD cohort, also supports the use of MT in CeAD patients with posterior circulation LVO. Consistent with the two previous analyses, recanalization rates and functional outcomes after MT were comparable to those of the general population [25, 28], despite the more frequent need for intra‐arterial drug administration in CeAD patients, which may attest to the higher complexity of the endovascular procedure in this vascular territory as well.

The strengths of our study include its large multicenter design, rigorous statistical adjustment, and the inclusion of a meta‐analysis, enhancing the robustness of our conclusions. However, some limitations need to be addressed. First, our control group consisted of non‐CeAD patients undergoing MT, meaning only indirect evaluation of MT efficacy was possible; obviously, it is not ethically feasible to withhold MT from CeAD patients with LVO‐AIS. Second, while our data were derived from multiple centers and collected prospectively, the retrospective design requires caution when interpreting the results. Third, although the management of LVO patients followed current guidelines, there was no standardized protocol across centers regarding procedural characteristics of MT or the management of CeAD patients. This inter‐center variability may introduce performance bias. Nevertheless, this aspect also represents a strength, as it mirrors real‐world practice: achieving similar outcomes despite differing protocols further strengthens the recommendation for intervention in CeAD patients. Fourth, the adverse events analyzed have been selected based on literature evidence identifying the most frequent MT‐related adverse events and on data availability, given the retrospective design. As a result, other potentially relevant safety outcomes may not have been captured or may differ between groups. Fifth, the present study did not specifically address the management of extracranial carotid lesions, which may introduce selection bias. Further studies evaluating internal carotid artery patency after emergent stenting and its association with functional outcomes are warranted. Lastly, the relatively small sample size of CeAD patients limits subgroup analyses, particularly for posterior circulation strokes.

## 5 Conclusions

According to this large multicenter study, despite some procedural challenges, EVT in CeAD patients achieves similar rates of optimal recanalization and favorable outcomes compared to matched patients with other stroke etiologies in both the anterior and posterior circulation, while demonstrating significantly lower mortality rates. These findings empower current recommendations to consider EVT for eligible patients with spontaneous CeAD.

## Author Contributions

**Rosario Pascarella:** writing – review and editing. **Luca Weis:** formal analysis, supervision, writing – review and editing, writing – original draft, software. **Mauro Gentile:** writing – review and editing. **Alessia Giossi:** writing – review and editing. **Michele Besana:** writing – review and editing. **Andrea Zini:** writing – review and editing, supervision. **Francesco Favruzzo:** conceptualization, investigation, writing – original draft, methodology, visualization, writing – review and editing, project administration, data curation, supervision. **Marialuisa Zedde:** supervision, writing – review and editing, data curation. **Stefano Vallone:** writing – review and editing. **Andrea Fiacca:** writing – review and editing. **Francesco Valletta:** writing – review and editing. **Manuel Cappellari:** writing – review and editing. **Benedetto Petralia:** writing – review and editing. **Iacopo Valente:** writing – review and editing. **Pietro Caliandro:** writing – review and editing. **Luigi Simonetti:** writing – review and editing. **Roberto Menozzi:** writing – review and editing. **Maria Giulia Mosconi:** writing – review and editing. **Francesco Causin:** writing – review and editing. **Aurelia Zauli:** writing – review and editing. **Maurizio Paciaroni:** writing – review and editing, supervision. **Chiara Ferraro:** writing – review and editing. **Paolo La Spina:** writing – review and editing. **Claudio Baracchini:** conceptualization, investigation, writing – original draft, methodology, visualization, validation, writing – review and editing, project administration, supervision. **Agostino Tessitore:** writing – review and editing. **Alessandro Pezzini:** writing – review and editing, supervision, data curation. **Ludovica Ferraù:** writing – review and editing.

## Funding

This study was supported by the author(s) disclosed no external funding.

## Conflicts of Interest

M.P. reports receiving personal honoraria for serving on the speaker bureaus of iRhythm, Daiichi Sankyo, and Boehringer Ingelheim. A.Z. reports receiving personal honoraria for serving on the speaker bureaus of Daiichi Sankyo, Astra Zeneca, Angels Initiative, and Pfizer, has received consulting fees from Boehringer Ingelheim, CSL Behring, and the Angels Initiative, has received payment for expert testimony from Alexion‐Astra Zeneca, and has received payment to participate on a data safety monitoring board or advisory board for Bayer, Astra Zeneca, and Boehringer Ingelheim. M.C. reports receiving personal honoraria for serving on the speaker bureaus of Daiichi Sankyo, Boehringer Ingelheim, AstraZeneca, and Pfizer, and has received payment to participate on a data safety monitoring board or advisory board for AstraZeneca and Boehringer Ingelheim.

## Supporting information


**Figure S1:** Comprehensive stroke centers participating in the study.
**Figure S2:** Study flowchart.
**Figure S3:** Pie charts illustrating the relative proportions of intracranial occlusion sites in the CeAD and non‐CeAD cohorts.
**Figure S4:** Covariate balance improvement between CeAD and Non‐CeAD groups before and after PSM Matching.
**Figure S5:** Age distribution of patients achieving a 90‐day mRS of 0–2 (A) and of those who died within 90 days (B) following MT treatment in CeAD and PSM‐matched non‐CeAD cohorts.
**Figure S6:** PRISMA flow chart of the meta‐analysis.
**Figure S7:** Traffic Light Plot showing the risk of bias for the seven different domains of each study included in the meta‐analysis.
**Figure S8:** Summary plot showing the overall risk of bias for the seven different domains of the studies included in the meta‐analysis.
**Table S1:** Factors associated with functional outcome in patients treated with MT.
**Table S2:** Results from adjusted multivariable logistic analyses showing the association of MT and outcomes in patients with spontaneous CeAD and overall patients with other stroke etiologies.
**Table S3:** Effect of ASPECTs and site of occlusion on functional outcomes.
**Table S4:** Baseline characteristics of CeAD and non‐CeAD patients with anterior and posterior LVO AIS.
**Table S5:** Functional and safety outcomes of sensitivity analysis for CeAD and matched non‐CeAD patients with anterior circulation LVO.

## Data Availability

The data that support the findings of this study are available from the corresponding author upon reasonable request.
