# Supplementary material for: Mechanical Thrombectomy in Patients With Cervical Artery Dissection: A Multicenter Analysis on Technical, Safety, and Functional Outcomes
Source: Eur J Neurol. 2026 Jun 22;33(6):e70632. doi: 10.1111/ene.70632 (PMC13284889; doi:10.1111/ene.70632)
Supplement: Supplementary file 1 — Figure S1: Comprehensive stroke centers participating in the study. Figure S2: Study flowchart. Figure S3: Pie charts illustrating the relative proportions of intracranial occlusion sites in the CeAD and non‐CeAD cohorts. Figure S4: Covariate balance improvement between CeAD and Non‐CeAD groups before and after PSM Matching. Figure S5: Age distribution of patients achieving a 90‐day mRS of 0–2 (A) and of those who died within 90 days (B) following MT treatment in CeAD and PSM‐matched non‐CeAD cohorts. Figure S6: PRISMA flow chart of the meta‐analysis. Figure S7: Traffic Light Plot showing the risk of bias for the seven different domains of each study included in the meta‐analysis. Figure S8: Summary plot showing the overall risk of bias for the seven different domains of the studies included in the meta‐analysis. Table S1: Factors associated with functional outcome in patients treated with MT. Table S2: Results from adjusted multivariable logistic analyses showing the association of MT and outcomes in patients with spontaneous CeAD and overall patients with other stroke etiologies. Table S3: Effect of ASPECTs and site of occlusion on functional outcomes. Table S4: Baseline characteristics of CeAD and non‐CeAD patients with anterior and posterior LVO AIS. Table S5: Functional and safety outcomes of sensitivity analysis for CeAD and matched non‐CeAD patients with anterior circulation LVO. [file ENE-33-e70632-s001.docx]

**Handling of missing data**
Missing values are reported in each table. The study sample was defined *a priori* as the set of patients with available prespecified variables (procedural adverse events and functional outcomes). Records lacking these variables were excluded from the final set. At the feature level, only variables with less than 50% missing data were included. To reduce imputation error and bias from excessive incompleteness, records with more than eight missing values out of 52 candidate covariates were excluded. Missingness mechanisms were assessed using the MissMech package in R. Missing data in covariates were imputed using the R missRanger algorithm (pmm.k =10, number of tree=1500), which accommodates both continuous and categorical variables.

Predictor variables were coded according to their measurement scale: nominal variables as factors and ordinal variables as ordered factors, preserving category-specific patterns. Continuous variables were imputed using predictive mean matching to minimize the influence of outliers. Imputation quality was evaluated using out-of-bag (OOB) error estimates, expressed as Normalized Root Mean Squared Error (NRMSE) for continuous variables and Proportion of Falsely Classified (PFC) entries for categorical variables. To maintain representativeness, artificial missingness for OOB evaluation was introduced proportionally to the original missingness level.

Among the 52 variables initially considered in this study - including clinical and demographic characteristics (n=11), risk factors (n=14), pharmacological therapy (n=7), procedural characteristics (n=10), adverse events (n=6), recanalization rates (n=1), and functional outcomes (n=3) - overall data completeness was high. One variable, “time to needle,” was excluded from analysis due to excessive missingness, with over 68% of values missing or not applicable (598 out of 1,861 cases). Of the remaining 51 variables, 75% (39/52) had less than 1% missing data. Specifically, 36 variables had complete data, 10 had 1–4% missingness, 3 had 5–9%, and 3 variables had more than 15% missing data. Missingness mechanisms were assessed using the MissMech package in R. The Hawkins test yielded a p-value of 6.2 × 10⁻²⁹¹, rejecting the assumption of multivariate normality and/or homoscedasticity. The nonparametric test also rejected the MCAR hypothesis (p = 1.3 × 10⁻¹⁰). These results suggest that missing data in the study were not *Missing Completely at Random* (MCAR), but more likely *Missing at Random* (MAR), thereby supporting the use of imputation techniques that leverage observed data patterns. Imputation quality, assessed with the out-of-bag (OOB) error estimates, showed an average normalized root mean square error (NRMSE) of 0.883 across continuous variables and an average proportion of falsely classified (PFC) of 0.162 across categorical variables, indicating satisfactory imputation accuracy while preserving the underlying distributions.

Names and affiliations of the DISSECT Study Group.

| **The Writing Committee for the DISSECT Study Group** |
| --- |
| Alvise Fattorello Salimbeni^1^ MD (a.fattorellosalimbeni@gmail.com), Alessandra Pes^1^ MD (alessandra.pes@studenti.unipd.it), Ludovica De Rosa^1^ MD (ludovica.derosa@studenti.unipd.it), Matteo Zaccagnino^1^ MD (matteo.zaccagnino@aopd.veneto.it), Federica Viaro^1^ MD (federico.viaro@aopd.veneto.it), Alessio Pieroni^1^ MD (alessio.pieroni@aopd.veneto.it), Stefano Mozzetta^1^ MD (stefano.mozzetta@aopd.veneto.it), Paola Castellini^2^ MD ([pcastellini@ao.pr.it](mailto:pcastellini@ao.pr.it)), Giorgia Abrignani^2^ MD ([gabrignani@ao.pr.it](mailto:gabrignani@ao.pr.it)), Lilia Latte^2^ MD ([llatte@ao.pr.it](mailto:llatte@ao.pr.it)), Maria Claudia Trapasso^2^ MD ([mtrapasso@ao.pr.it](mailto:mtrapasso@ao.pr.it)), Antonio Genovese^2^ MD ([agenovese@ao.pr.it](mailto:agenovese@ao.pr.it)), Lorenzo Corradi^3^ ([lorenzo.corradi@unipr.it](mailto:lorenzo.corradi@unipr.it)), Giulia Capurri^4^ MD ([gcapurri@ao.pr.it](mailto:gcapurri@ao.pr.it)), Roberta Partesano^4^ MD ([rpartesano@ao.pr.it](mailto:rpartesano@ao.pr.it)), Enrico Epifani^4^ MD ([eepifani@ao.pr.it](mailto:eepifani@ao.pr.it)), Marta Garbuglia^5^ MD ([marta.garbuglia@guest.policlinicogemelli.it](mailto:marta.garbuglia@guest.policlinicogemelli.it)), Ludovica Migliaccio^6^ MD (ludovica.migliaccio3@unibo.it) |
| **Affiliations of The Writing Committee for the DISSECT Study Group:** |
| ^1^ Department of Neurology and Stroke Center, Azienda Ospedaliera Padova, University of Padua, Padova 35128 Italy;  ^2^ Department of Emergency, Stroke Care Program, Parma University Hospital, Parma 43126 IT;  ^3^ Department of Medicine and Surgery, University of Parma, Parma 43126 IT;  ^4^ Neuroradiology Department, Parma University Hospital, Parma 43126 IT;  ^5^ Department of Neurology and Stroke Center, Fondazione Policlinico Universitario A. Gemelli IRCCS, Rome 00136 IT;  ^6^ Department of Neurosciences, Università Cattolica del Sacro Cuore, Rome 00136 IT;  ^7^ Department of Neurology and Stroke Center, IRCCS Istituto Delle Scienze Neurologiche Di Bologna, Maggiore Hospital, Bologna 40133 IT; |


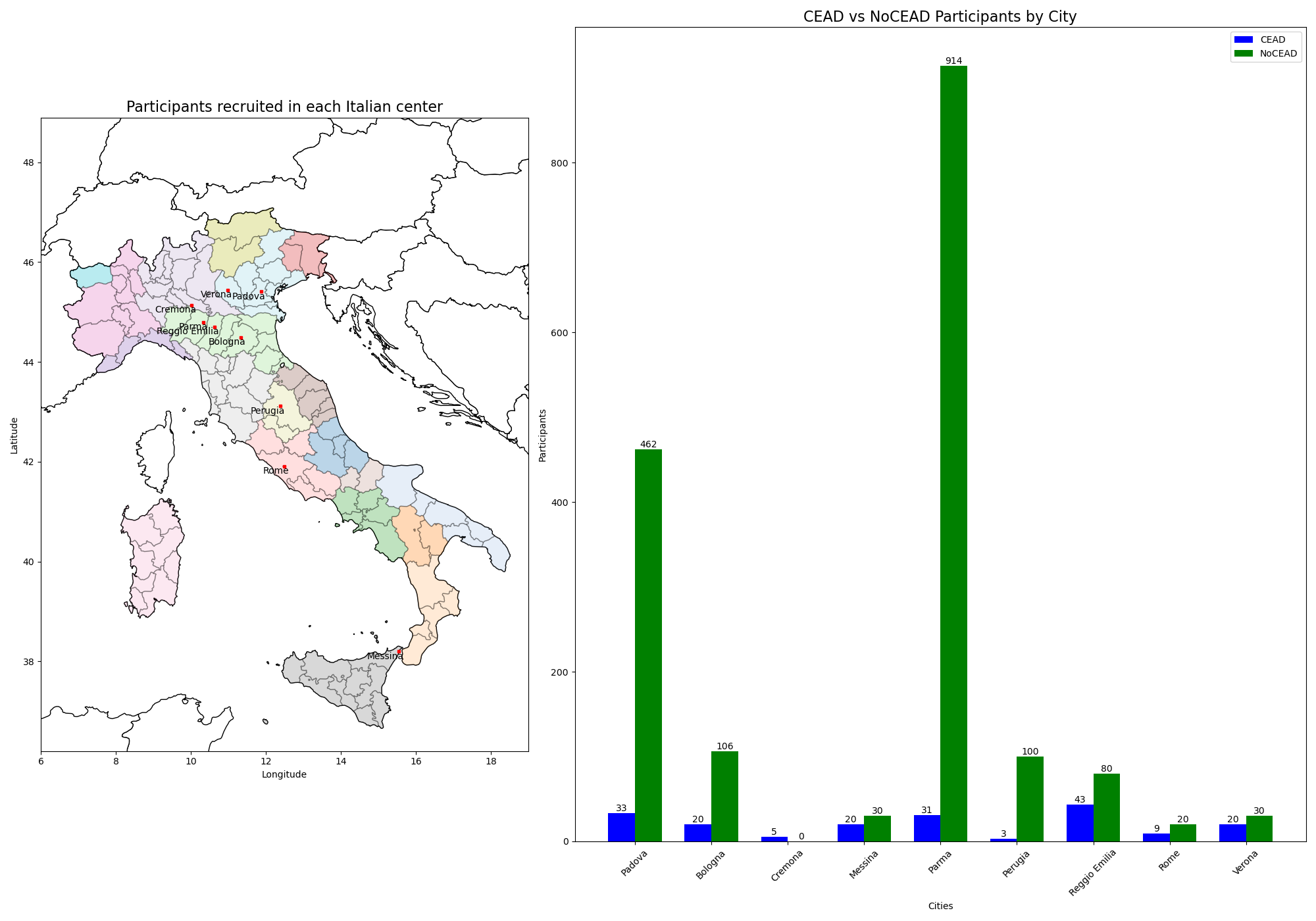


**eFigure 1** - Comprehensive stroke centers participating to the study.

**eTable 1** – Factor associated with functional outcome in patients treated with MT.

IQR: interquartile range; MT: mechanical thrombectomy; ASPECTS: Alberta Stroke Program Early CT score; NIHSS: National Institute of Health Stroke Scale; mRS: modified Rankin Scale.

**eFigure2 -** Study flow chart

**eFigure3 –** Pie charts illustrating the relative proportions of intracranial occlusion sites in the CeAD and non-CeAD cohorts.

ICA: internal carotid artery; MCA: middle cerebral artery; BA: basilar artery; VA: vertebral artery; PCA: posterior cerebral artery; ACA: anterior cerebral artery.

**
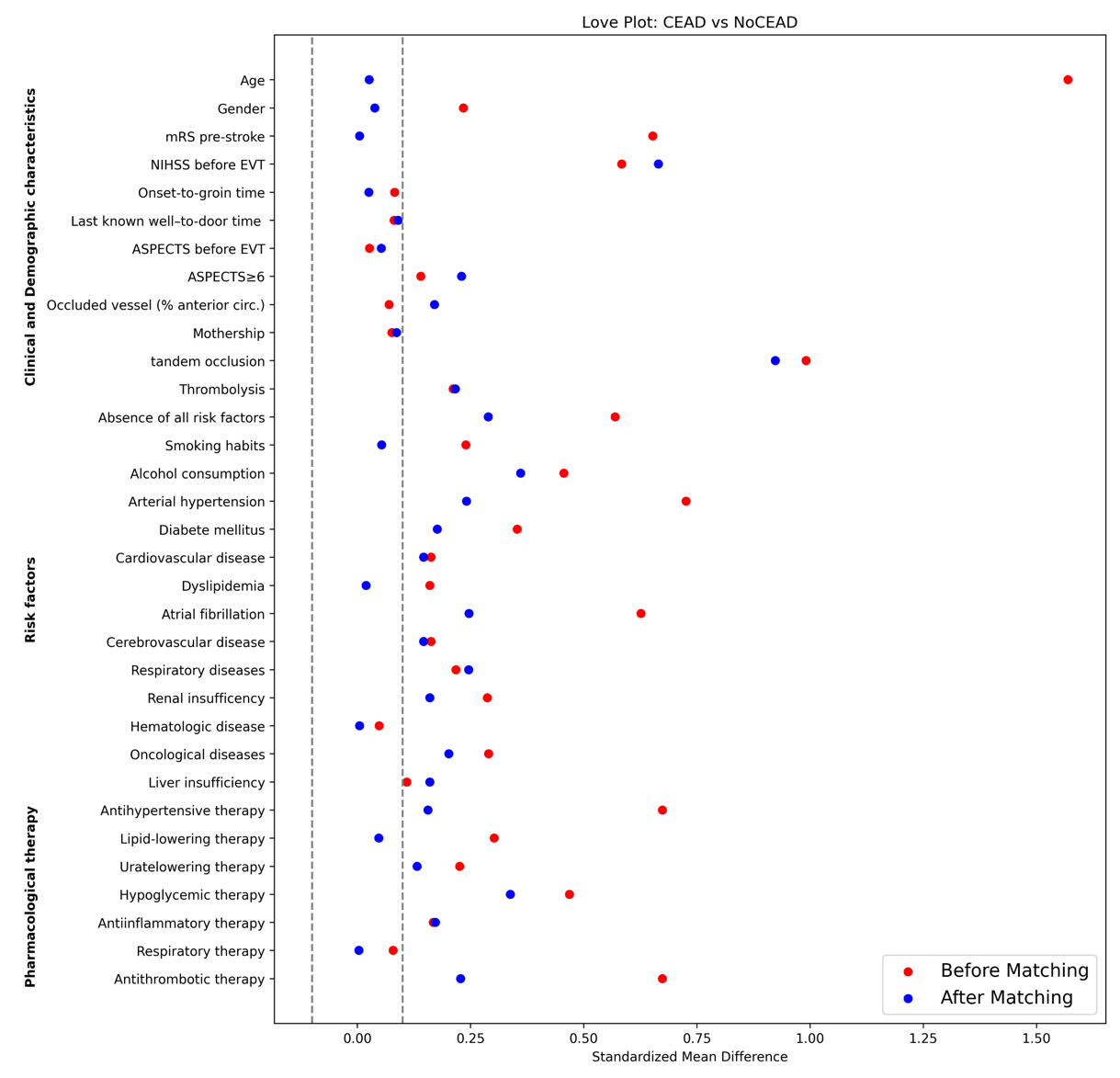
**

**eFigure4 -** Covariate balance improvement between CeAD and Non-CeAD groups before and after PSM Matching.

Standardized mean differences (SMDs) for clinical, demographic, risk factor, and pharmacological therapy variables are shown before (red) and after (blue) propensity score matching. Covariates are grouped by domain. Vertical dashed lines at ±0.1 indicate the commonly accepted threshold for acceptable balance. Matching substantially improved covariate balance across domains.

**eTable 2** - Results from adjusted multivariable logistic analyses showing the association of MT and outcomes in patients with spontaneous CeAD and overall patients with other stroke etiologies.

Note: multivariable logistic regression and ANCOVA models were adjusted for age, baseline mRS, gender, baseline NIHSS, absence of risk factor, smoking habits, arterial hypertension, diabetes mellitus, atrial fibrillation, antihypertensive therapy, hypoglycemic therapy and antithrombotic therapy.

IQR: interquartile range; MT: mechanical thrombectomy; mTICI: modified treatment in cerebral infarction; NIHSS: National Institute of Health Stroke Scale; mRS: modified Rankin Scale; OR: odd ratio.


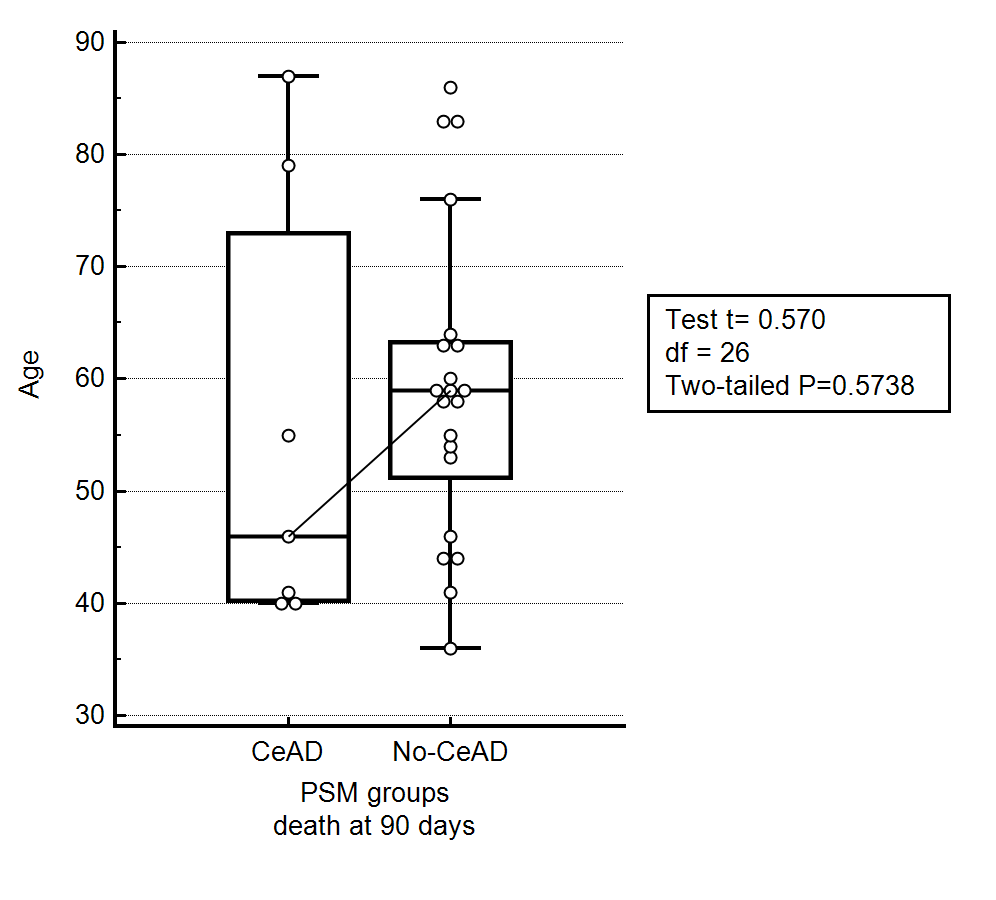

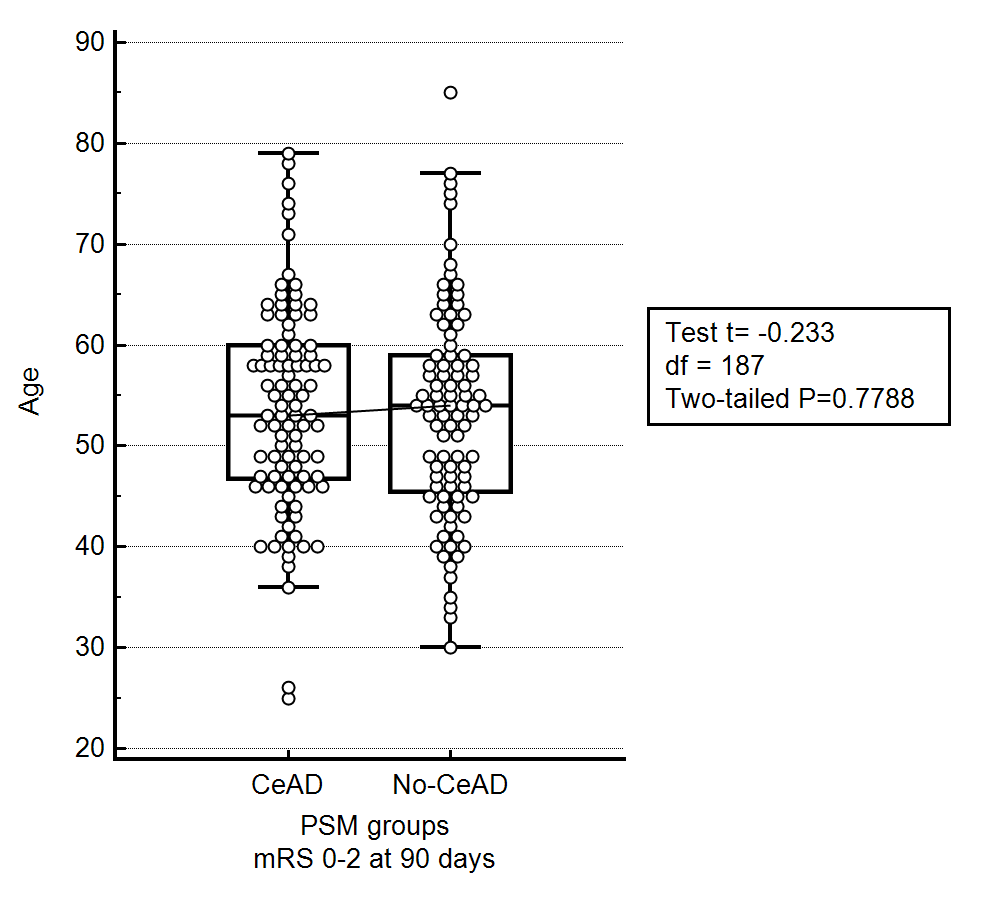


A

B

**eFigure5 -** Age distribution of patients achieving a 90‑day mRS of 0–2 (A) and of those who died within 90 days (B) following MT treatment in CeAD and PSM-matched non CeAD cohorts.

**eTable 3 – Effect of ASPECTs and site of occlusion on functional outcomes.**

Note: Overall and PSM–matched functional outcomes comparisons between CeAD and non-CeAD subgroups. For Raw comparison Mann whitney –U Test and chi-squared were adopted. ***** Multivariable logistic regression and Ancova models were adjusted for Age, Baseline mRS, Gender, baseline NIHSS, absence of risk factor, Smoking habits, Arterial hypertension, Diabetes mellitus, Atrial fibrillation, Antihypertensive therapy, Hypoglycemic therapy and Antithrombotic therapy; **&**: sensitivity analysis adjusting also for anatomical sites of occlusion (ACA, ICA, MCA,BA,VA,PCA) to previous covariates; **£**: sensitivity analysis adjusting also for ASPECTS.

IQR: interquartile range; mRS: modified Rankin Scale; OR: odd ratio.

**eTable 4** - Baseline characteristics of CeAD and non-CeAD patients with anterior and posterior LVO AIS.

mRS: modified Rankin Scale; NIHSS: National Institute of Health Stroke Scale; ASPECTS: Alberta Stroke Program Early CT score; IQR: interquartile range

**eTable 5 -** Functional and safety outcomes of sensitivity analysis for CeAD and matched non-CeAD patients with anterior circulation LVO

Note: * multivariable logistic regression and Ancova models were adjusted for Age, Baseline mRS, Gender, baseline NIHSS, absence of risk factor, Smoking habits, Arterial hypertension, Diabetes mellitus, Atrial fibrillation, Antihypertensive therapy, Hypoglycemic therapy and Antithrombotic therapy was used to compare CeAD vs. PSM matched non CeAD.

IQR: interquartile range; MT: mechanical thrombectomy; mTICI: modified treatment in cerebral infarction; NIHSS: National Institute of Health Stroke Scale; mRS: modified Rankin Scale; OR: odd ratio.


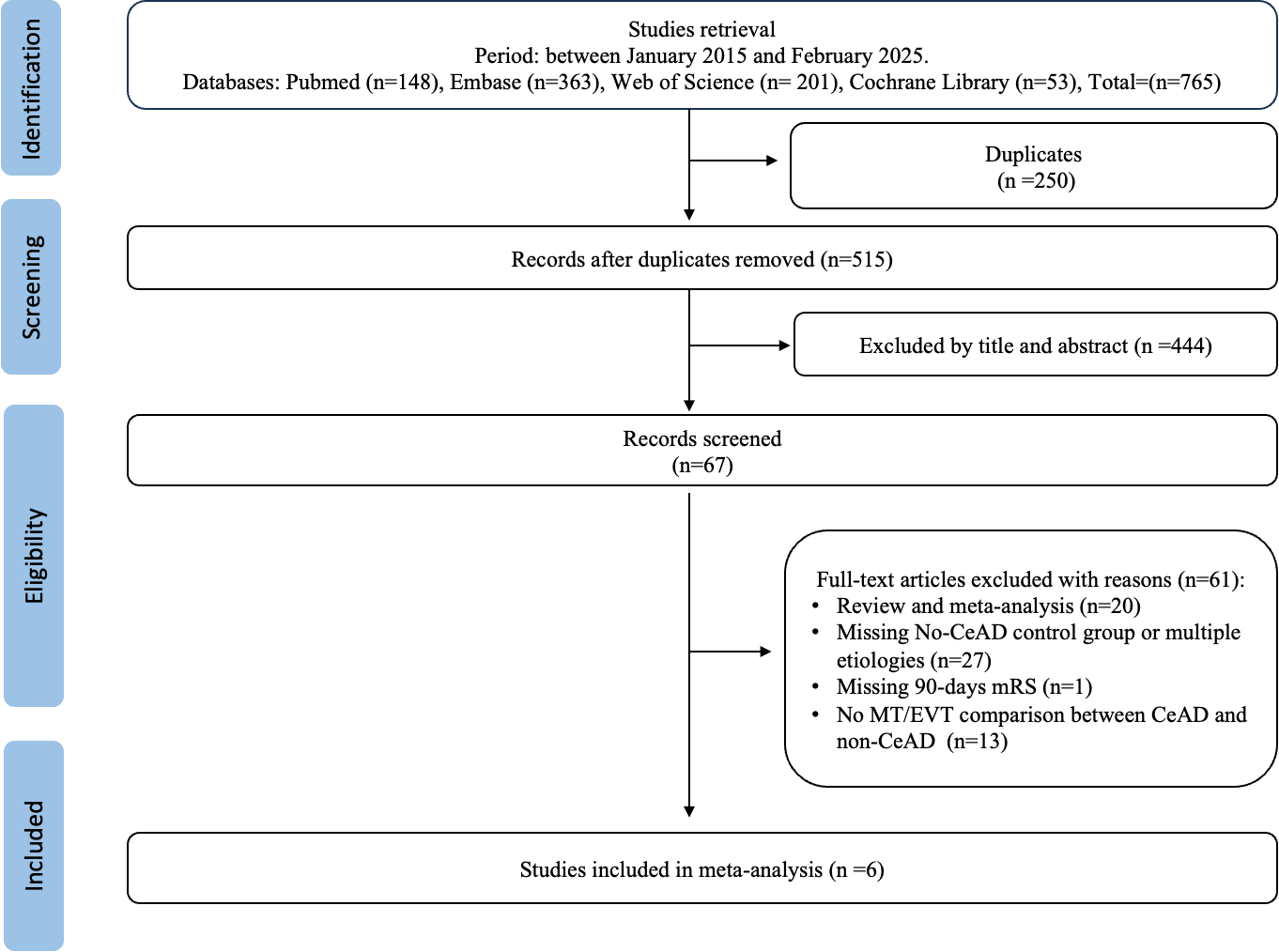


**eFigure6 –** PRISMA flow chart of the meta-analysis

**eFigure7 -** Traffic Light Plot showing the risk of bias for the seven different domains of each study included in the meta-analysis


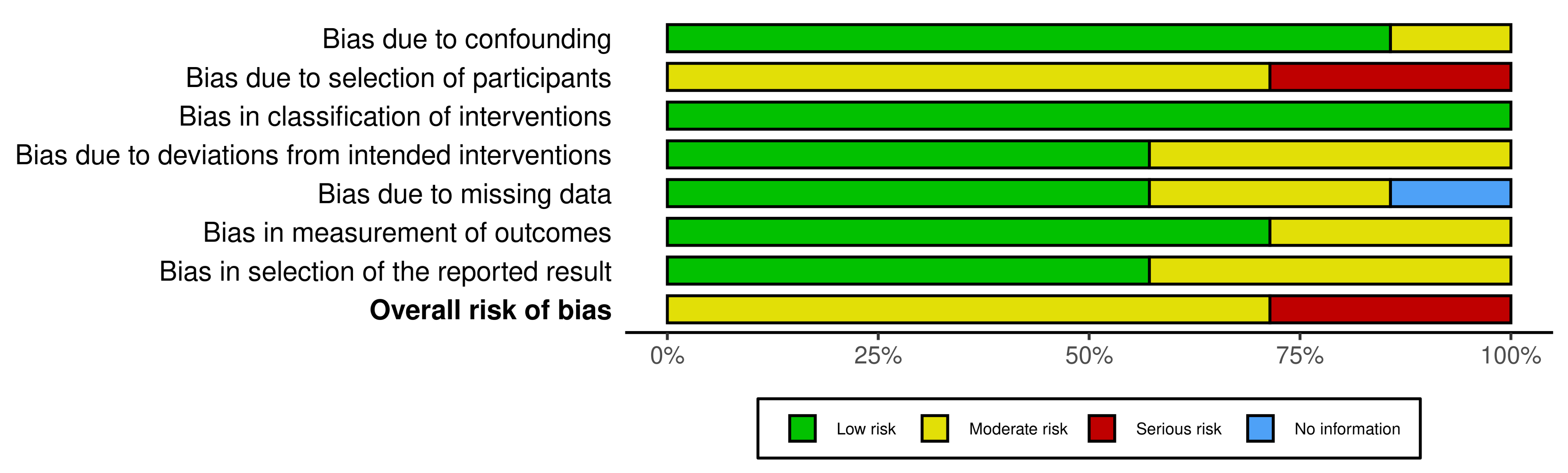


**eFigure8 –** Summary plot showing the overall risk of bias for the seven different domains of the studies included in the meta-analysis.

**Definitions of adverse events related to MT**

Adverse events associated with mechanical thrombectomy were defined for this study as follows, based on prior clinical trial protocols and registry reports in the acute ischemic stroke literature:

1. Symptomatic intracranial hemorrhage (sICH): any intracranial hemorrhage on post-procedure imaging (parenchymal hematoma PH1/PH2, remote ICH, SAH or IVH) associated with neurological deterioration defined as an increase of ≥4 points on the NIHSS, or hemorrhage judged to be the principal cause of death.^1^
2. Asymptomatic intracranial hemorrhage (aICH): any intracranial hemorrhage on post-procedural imaging (parenchymal hematoma PH1/PH2, remote ICH, SAH or IVH) that is not associated with NIHSS worsening ≥4 points.^2^
3. Embolization to distal territory (EDT) / distal emboli: new intraprocedural emboli within the originally affected vascular territory (distal to the original occlusion), usually documented angiographically or on early post-procedural diffusion MRI.^3^
4. Iatrogenic dissection: new angiographic evidence of intimal flap, double lumen, or intramural hematoma on procedural or follow-up vascular imaging, recorded when temporally related to MT maneuvers.^4^
5. Vasospasm: reversible arterial narrowing of ≥20% but <80% of the normal vessel diameter, detected on digital subtraction angiography (DSA) immediately following mechanical irritation (e.g., stent-retriever deployment or aspiration).^5^
6. Re-occlusion: reappearance of occlusion in a previously successfully recanalized artery within 24 hours, following an initial restoration of antegrade flow.^6^
7. Vessel perforation: an intraprocedural breach of an intracranial arterial wall resulting in active contrast extravasation on angiography, typically visualized during digital subtraction angiography (DSA) and indicating disruption of the vessel integrity.^7^

**DATA EXTRACTION**

The following data were retrieved from each site and analyzed centrally: baseline demographic (age, gender), clinical [pre-stroke modified Rankin Scale (mRS), National Institute of Health Stroke Scale (NIHSS) score, history of arterial hypertension, diabetes mellitus, or hypercholesterolemia, smoking habit, alcohol intake (heavy consumption was defined as > 14 drinks for men and > 7 drinks for women per week), history of coronary syndromes, previous or current atrial fibrillation, previous cerebrovascular events (TIA, ischemic or hemorrhagic stroke), chronic respiratory disease, thrombotic or bleeding diathesis, chronic kidney disease, chronic liver disease, active cancer], pharmacological (anti-thrombotic, anti-hypertensive, lipid-lowering, urate-lowering, anti-diabetic and anti-inflammatory drugs; intravenous thrombolysis), neuroradiological [baseline Alberta Stroke Program Early CT Score (ASPECTS), LVO site, tandem occlusion defined as a simultaneous presence of cervical vessel occlusion [eg. internal carotid artery (ICA)] and proximal large vessel occlusion (eg. origin of the middle cerebral artery)], and procedural characteristics [type of anesthesia (conscious sedation, general anesthesia), type of technique adopted (thromboaspiration, mechanical thrombectomy), numbers of attempts, medications used during MT, extracranial stenting], time intervals (last-know-well to door, door to needle, door to groin, groin to recanalization), treatment paradigm (mothership, drip-and-ship), recanalization rates [modified Treatment in Cerebral Infarction (mTICI) scale], MT-related adverse events (iatrogenic dissection, distal embolization, vasospasm, intracranial hemorrhage, re-occlusion of intracranial recanalized vessel, vessel perforation), functional outcomes (mRS at 90 days, mortality at discharge and at 90 days), and presumed stroke etiology.

**Meta-analysis full electronic search strategy**

**MEDLINE** via **PubMed** (searched 15 Feb 2025), <https://pubmed.ncbi.nlm.nih.gov/advanced/>, **Platform/Database:** PubMed (MEDLINE), **Date range:** 01 Jan 2015 – 15 Feb 2025, **Limits:** Humans; English; Adult (MeSH or text), **Fields:** MeSH + Title/Abstract (tiab).

(

  ("Stroke"[Mesh] OR "Brain Ischemia"[Mesh] OR "Cerebral Infarction"[Mesh]

   OR stroke*[tiab] OR "ischaemic stroke"[tiab] OR "ischemic stroke"[tiab]

   OR "brain infarct*"[tiab] OR "cerebral infarct*"[tiab] OR "brain ischem*"[tiab]

   OR "cerebrovascular accident*"[tiab] OR CVA[tiab] OR "cerebrovascular disorder*"[tiab]

  )

  AND

  ("Dissection"[Mesh] OR dissection*[tiab]

   OR "cervical artery dissection"[tiab] OR "carotid dissection"[tiab]

   OR "carotid artery dissection"[tiab] OR "vertebral dissection"[tiab]

   OR "vertebral artery dissection"[tiab] OR CeAD[tiab] OR CAD[tiab]

  )

  AND

  ("Endovascular Procedures"[Mesh] OR "Thrombectomy"[Mesh]

   OR endovascular[tiab] OR "endovascular treatment"[tiab] OR EVT[tiab]

   OR thrombectom*[tiab] OR "endovascular thrombectomy"[tiab]

   OR "mechanical thrombectomy"[tiab] OR "stent retriever"[tiab]

   OR aspiration[tiab] OR "endovascular surgery"[tiab]

  )

)

AND ("2015/01/01"[Date - Publication] : "2025/02/15"[Date - Publication])

AND Humans[Mesh]

AND English[lang]

AND ("Adult"[Mesh] OR adult*[tiab])

**Embase** via Ovid (searched 15 feb 2025), <https://ovidsp.dc1.ovid.com/ovid-new-a/ovidweb.cgi>, **Platform/Database:** Ovid Embase (Emtree controlled vocabulary)
**Date range:** 01 Jan 2015 – 15 Feb 2025, **Limits:** Humans; Adult; English; Year 2015–Current (up to 15 Feb 2025), **Fields:** .ti,ab,kw (title/abstract/author keywords); controlled vocabulary uses /exp, **Approach:** Emtree (exploded) + free-text (ti,ab,kw) + Boolean operators; limits applied after combining concepts.

1. exp stroke/ or exp brain ischemia/ or exp cerebral infarction/

2. (stroke* or ischemic stroke or ischaemic stroke or brain infarct* or cerebral infarct* or brain ischem* or cerebrovascular accident* or CVA or cerebrovascular disorder*).ti,ab,kw.

3. 1 or 2

4. exp artery dissection/ or exp carotid artery dissection/ or exp vertebral artery dissection/

5. (dissection* or cervical artery dissection or carotid dissection or carotid artery dissection or vertebral dissection or vertebral artery dissection or CeAD or CAD).ti,ab,kw.

6. 4 or 5

7. exp endovascular procedure/ or exp thrombectomy/ or exp mechanical thrombectomy/

8. (endovascular or endovascular treatment or EVT or thrombectom* or endovascular thrombectomy or mechanical thrombectomy or stent retriever or aspiration or endovascular surgery).ti,ab,kw.

9. 7 or 8

10. 3 and 6 and 9

11. limit 10 to (human and adult and english language)

12. limit 11 to yr="2015 -Current"

**Web of Science Core Collection** (searched 15 Feb 2025), **Platform/Database:** Web of Science Core Collection, **Date range:** 2015–2025 (up to 15 Feb 2025), **Limits/Refinements:** English; (optional) Article/Review, **Fields:** Topic (TS) searches title/abstract/keywords.

TS=(

  (stroke* OR "ischemic stroke" OR "ischaemic stroke" OR "brain infarct*" OR "cerebral infarct*"

   OR "brain ischem*" OR "cerebrovascular accident*" OR CVA OR "cerebrovascular disorder*")

  AND

  (dissection* OR "cervical artery dissection" OR "carotid dissection" OR "carotid artery dissection"

   OR "vertebral dissection" OR "vertebral artery dissection" OR CeAD OR CAD)

  AND

  (endovascular OR "endovascular treatment" OR EVT OR thrombectom* OR "endovascular thrombectomy"

   OR "mechanical thrombectomy" OR "stent retriever" OR aspiration OR "endovascular surgery")

)

Refine by: Languages = English

Timespan: 2015–2025 (search date: 15 Feb 2025)

Indexes: Web of Science Core Collection

**References**

1. Saver Jeffrey L., Goyal Mayank, Bonafe Alain, et al. Stent-Retriever Thrombectomy after Intravenous t-PA vs. t-PA Alone in Stroke. *N Engl J Med*. 2015;372(24):2285-2295. doi:10.1056/NEJMoa1415061

2. van der Steen W, van der Ende NAM, Luijten SPR, et al. Type of intracranial hemorrhage after endovascular stroke treatment: association with functional outcome. *J Neurointerventional Surg*. 2023;15(10):971-976. doi:10.1136/jnis-2022-019474

3. Yeo LLL, Holmberg A, Mpotsaris A, et al. Posterior Circulation Occlusions May Be Associated with Distal Emboli During Thrombectomy : Factors for Distal Embolization and a Review of the Literature. *Clin Neuroradiol*. 2019;29(3):425-433. doi:10.1007/s00062-018-0679-z

4. Goeggel Simonetti B, Hulliger J, Mathier E, et al. Iatrogenic Vessel Dissection in Endovascular Treatment of Acute Ischemic Stroke. *Clin Neuroradiol*. 2019;29(1):143-151. doi:10.1007/s00062-017-0639-z

5. Uchikawa H, Kuroiwa T, Nishio A, et al. Vasospasm as a major complication after acute mechanical thrombectomy with stent retrievers. *J Clin Neurosci Off J Neurosurg Soc Australas*. 2019;64:163-168. doi:10.1016/j.jocn.2019.03.011

6. Marto JP, Strambo D, Hajdu SD, et al. Twenty-Four-Hour Reocclusion After Successful Mechanical Thrombectomy: Associated Factors and Long-Term Prognosis. *Stroke*. 2019;50(10):2960-2963. doi:10.1161/STROKEAHA.119.026228

7. Schulze-Zachau V, Rommers N, Ntoulias N, et al. Insights into vessel perforations during thrombectomy: Characteristics of a severe complication and the effect of thrombolysis. *Eur Stroke J*. 2025;10(1):63-73. doi:10.1177/23969873241272542
